# Um Novo Vislumbre da Radiografia de Tórax: Revelando o Significado das Calcificações do Arco Aórtico na Recorrência de Acidente Vascular Cerebral

**DOI:** 10.36660/abc.20240404

**Published:** 2024-08-07

**Authors:** José Ribeiro

**Affiliations:** 1 Unidade Local de Saúde de Gaia e Espinho Vila Nova de Gaia Portugal Serviço de Cardiologia, Unidade Local de Saúde de Gaia e Espinho, Vila Nova de Gaia – Portugal

**Keywords:** Radiografia Pulmonar de Massa, Aorta Torácica, Acidente Vascular Cerebral, Fatores de Risco de Doenças Cardíacas, Diagnóstico por Imagem

O impacto global das doenças cardiovasculares continua a ser significativo, apesar das melhorias no diagnóstico e tratamento. A importância epidemiológica e os resultados clínicos associados enfatizam a importância da estratificação de risco e das medidas preventivas.

No domínio do diagnóstico médico, cada observação sutil tem o potencial de desbloquear conhecimentos cruciais sobre o prognóstico e a gestão de várias condições de saúde. Uma dessas observações que tem chamado a atenção é a presença de calcificação no arco aórtico detectada na radiografia de tórax. Embora tradicionalmente vista como um marcador de risco cardiovascular, estudos recentes desencadearam um debate sobre se a calcificação do arco aórtico (CAA) poderia servir como um preditor independente de AVC recorrente.

A calcificação aórtica, especialmente em seu segmento descendente, está associada à ocorrência de acidente vascular cerebral, como mostrado no estudo Heinz Nixdorf Recall de base populacional.^1^ Tian et al. investigaram a CAA medida por radiografia de tórax em 27.166 adultos chineses com mais de 50 anos, descobrindo que a CAA é útil para estratificação de risco cardiovascular.^2^ Da mesma forma, Iribarren et al. estudaram CAA em uma coorte americana de 116.309 pacientes, predominantemente caucasianos, encontrando um risco aumentado de hospitalização ou morte durante um acompanhamento médio de 28 anos.^3^ Outros estudos mostraram correlações entre CAA e fatores de risco convencionais.^4–6^

Pesquisas recentes indicam que os mecanismos subjacentes ao acidente vascular cerebral embólico de origem indeterminada incluem doenças cardíacas de baixo risco embólico, embolias cerebrais paradoxais, lesões aórticas e doença arterial carotídea leve a moderada.^7^ K. Iijima et al. descobriram que a avaliação da CAA por meio de radiografia de tórax fornece informações benéficas para prever eventos cardiovasculares além dos fatores de risco tradicionais.^8^

Apesar dessas descobertas, desafios e controvérsias cercam o conceito de CAA como preditor de AVC recorrente. As críticas incluem a natureza retrospectiva de muitos estudos, potenciais variáveis de confusão e uma compreensão incompleta dos mecanismos precisos que ligam a CAA à recorrência do AVC.

Um estudo de coorte prospectivo, incluindo 203 pacientes que tiveram seu primeiro acidente vascular cerebral, também está sendo publicado nesta revista.^9^ Os autores dividiram os pacientes em dois grupos com base na incidência de acidentes cerebrovasculares recorrentes e os acompanharam por um ano. A CAA foi avaliada por radiografia de tórax e classificada em quatro categorias. Destes pacientes, 49 apresentaram recorrência do AVC no primeiro ano. A presença de CAA (≥ grau 1) e largura de distribuição de glóbulos vermelhos (RDW) foram significativamente associadas ao desenvolvimento de AVC recorrente.

Embora a radiografia seja um estudo de rotina, a aplicação de um escore de cálcio é demorada. Nesse contexto, a inteligência artificial (IA) pode se tornar uma ferramenta útil, mostrando efeitos satisfatórios em 5 tipos de calcificação vascular (coronária, aorta torácica, aorta abdominal, carótida e mama). A IA auxilia os radiologistas no diagnóstico de calcificação vascular com triagem preliminar e acelerando a eficiência do trabalho.^10,11^

A implementação de sistemas de apoio ao diagnóstico baseados em IA sublinha a importância da validação de vários parâmetros baseados em imagens. À medida que estes sistemas de IA se tornam mais integrados na prática clínica, a necessidade de evidências robustas para apoiar as suas recomendações torna-se cada vez mais crucial.

Enquanto o debate continua, os médicos enfrentam a tarefa de integrar estes resultados na sua prática clínica. A CAA deve ser considerada rotineiramente em algoritmos de estratificação de risco para AVC recorrente? Como essas informações podem influenciar as decisões de tratamento e estratégias preventivas? Essas questões destacam a necessidade de estudos multicêntricos prospectivos com amostras maiores para validar o valor preditivo da CAA e seu papel na patogênese do AVC.

Em conclusão, a noção de CAA como um preditor independente de AVC recorrente representa uma interseção fascinante da medicina cardiovascular e cerebrovascular. Embora estudos recentes sugiram uma associação potencial, são necessárias mais pesquisas para esclarecer os mecanismos subjacentes e estabelecer a sua utilidade clínica. Entretanto, os médicos devem continuar a avaliar os fatores de risco tradicionais e considerar a CAA como parte de uma avaliação abrangente do risco de AVC. Somente através da investigação científica contínua poderemos avançar na nossa compreensão e, em última análise, melhorar os resultados dos pacientes no domínio das doenças cerebrovasculares.

## References

[1] Hermann DM, Lehmann N, Gronewold J, Bauer M, Mahabadi AA, Weimar C (2015). Thoracic Aortic Calcification is Associated with Incident Stroke in the General Population in Addition to Established Risk Factors. Eur Heart J Cardiovasc Imaging.

[2] Tian WB, Zhang WS, Jiang CQ, Liu XY, Jin YL, Lam TH (2022). Aortic Arch Calcification and Risk of All-cause Mortality and Cardiovascular Disease: The Guangzhou Biobank Cohort Study. Lancet Reg Health West Pac.

[3] Iribarren C (1936). Calcification of the Aortic Valve. Am Heart J.

[4] Adar A, Onalan O, Cakan F, Akbay E, Karakaya E (2020). Aortic Arch Calcification on Routine Chest Radiography is Strongly and Independently Associated with Non-Dipper Blood Pressure Pattern. Arq Bras Cardiol.

[5] Desai MY, Cremer PC, Schoenhagen P (2018). Thoracic Aortic Calcification: Diagnostic, Prognostic, and Management Considerations. JACC Cardiovasc Imaging.

[6] Póvoa R (2020). Aortic Arch Calcification on routine Chest Radiography is Strongly and Independently Associated with Non-Dipper Blood Pressure Pattern. Arq Bras Cardiol.

[7] Kamel H, Pearce LA, Ntaios G, Gladstone DJ, Perera K, Roine RO (2020). Atrial Cardiopathy and Nonstenosing Large Artery Plaque in Patients With Embolic Stroke of Undetermined Source. Stroke.

[8] Iijima K, Hashimoto H, Hashimoto M, Son BK, Ota H, Ogawa S (2010). Aortic arch Calcification Detectable on Chest X-ray is a Strong Independent Predictor of Cardiovascular Events Beyond Traditional Risk Factors. Atherosclerosis.

[9] Çakan F, Sunal AS, Adar A, Onalan O (2024). A Calcificação do Arco Aórtico Observada na Radiografia de Tórax Pode Servir Como um Preditor Independente de Acidente Vascular Cerebral Recorrente. Arq Bras Cardiol.

[10] Kamel PI, Yi PH, Sair HI, Lin CT (2021). Prediction of Coronary Artery Calcium and Cardiovascular Risk on Chest Radiographs Using Deep Learning. Radiol Cardiothorac Imaging.

[11] Zhong Z, Yang W, Zhu C, Wang Z (2023). Role and Progress of Artificial Intelligence in Radiodiagnosing Vascular Calcification: A Narrative Review. Ann Transl Med.

