# Supplementary material for: Um Novo Vislumbre da Radiografia de Tórax: Revelando o Significado das Calcificações do Arco Aórtico na Recorrência de Acidente Vascular Cerebral
Source: Arq Bras Cardiol. 2024 Aug 7;121(7):e20240404. [Article in Portuguese] doi: 10.36660/abc.20240404 (PMC11495814; doi:10.36660/abc.20240404)
Supplement: Translation

# Translation: A Fresh Glimpse on Chest X-Ray: Revealing Significance of Aortic Arch Calcifications for Stroke Recurrence

**DOI:** 10.36660/abc.20240404i

**Authors:** José Ribeiro

**Affiliations:** 1 Vila Nova de Gaia Portugal Serviço de Cardiologia, Unidade Local de Saúde de Gaia e Espinho, Vila Nova de Gaia – Portugal

**Keywords:** Mass Chest X-Ray, Thoracic Aorta, Stroke, Heart Disease Risk Factors, Diagnostic Imaging

The global impact of cardiovascular disease continues to be significant, despite improvements in diagnosis and treatment. The epidemiological significance and associated clinical outcomes emphasize the importance of risk stratification and preventive measures.

In the realm of medical diagnostics, every subtle observation holds the potential to unlock crucial insights into the prognosis and management of various health conditions. One such observation that has garnered attention is the presence of calcification in the aortic arch as detected on chest X-ray. While traditionally viewed as a marker of cardiovascular risk, recent studies have ignited a debate on whether aortic arch calcification (AAC) could serve as an independent predictor of recurrent stroke.

Aortic calcification, especially in its descending segment, is associated with incident stroke, as shown in the population-based Heinz Nixdorf Recall study.^1^ Tian et al. investigated AAC measured by chest X-ray in 27,166 Chinese adults over 50 years, finding that AAC is useful for cardiovascular risk stratification.^2^ Similarly, Iribarren et al. studied AAC in an American cohort of 116,309 patients, predominantly Caucasians, finding an increased risk of hospitalization or death over a median follow-up of 28 years.^3^ Other studies have shown correlations between AAC and conventional risk factors.^4–6^

Recent research indicates that the mechanisms underlying embolic stroke of undetermined source include low-embolic risk cardiac diseases, paradoxical brain embolisms, aortic lesions, and mild-to-moderate carotid arterial disease.^7^ K. Iijima et al. found that AAC assessment via chest X-ray provides beneficial information for predicting cardiovascular events beyond traditional risk factors.^8^

Despite these findings, challenges and controversies surround the concept of AAC as a predictor of recurrent stroke. Criticisms include the retrospective nature of many studies, potential confounding variables, and an incomplete understanding of the precise mechanisms linking AAC to stroke recurrence.

A prospective cohort study, including 203 patients experiencing their first stroke event, is also being published in this journal.^9^ The authors divided the patients into two groups based on the incidence of recurrent cerebrovascular accidents and followed them up for one year. AAC was assessed by chest X-ray and classified into four categories. Of these patients 49 experienced recurrence of stroke within the first year. The presence of AAC (≥ grade 1) and red cell distribution width (RDW) were significantly associated with the development of recurrent stroke.

Although X-ray is a routine study, applying a calcium score is time-consuming. In this context, artificial intelligence (AI) may become a helpful tool, showing satisfactory effects in 5 types of vascular calcification (coronary, thoracic aorta, abdominal aorta, carotid, and breast). AI assists radiologists in diagnosing vascular calcification with preliminary screening and speeding up work efficiency.^10,11^

The implementation of AI-based diagnostic support systems underscores the importance of validating various imaging-based parameters. As these AI systems become more integrated into clinical practice, the need for robust evidence to support their recommendations becomes increasingly crucial.

While the debate continues, clinicians are faced with the task of integrating these findings into their clinical practice. Should AAC be routinely considered in risk stratification algorithms for recurrent stroke? How might this information influence treatment decisions and preventive strategies? These questions highlight the need for prospective multicenter studies with larger sample sizes to validate AAC's predictive value and its role in stroke pathogenesis.

In conclusion, the notion of AAC as an independent predictor of recurrent stroke represents a fascinating intersection of cardiovascular and cerebrovascular medicine. While recent studies suggest a potential association, further research is warranted to clarify the underlying mechanisms and establish its clinical utility. In the interim, clinicians should continue assessing traditional risk factors and consider AAC as part of a comprehensive stroke risk evaluation. Only through continued scientific inquiry can we advance our understanding and ultimately improve patient outcomes in the realm of cerebrovascular disease.
